# A Randomized, Placebo‐Controlled Crossover Study with Dipyridamole for Restless Legs Syndrome

**DOI:** 10.1002/mds.28668

**Published:** 2021-06-17

**Authors:** Diego Garcia‐Borreguero, Celia Garcia‐Malo, Juan José Granizo, Sergi Ferré

**Affiliations:** ^1^ Sleep Research Institute Madrid Spain; ^2^ Department of Clinical Epidemiology Hospital Universitario Infanta Cristina, Instituto de Investigaciones Sanitarias Puerta de Hierro Madrid Spain; ^3^ Integrative Neurobiology Section National Institute on Drug Abuse, Intramural Research Program, National Institutes of Health Baltimore Maryland USA

**Keywords:** clinical trial, adenosine ENT inhibitors, glutamate, dopamine agonists, restless legs syndrome (RLS)

## Abstract

**Background:**

New pharmacological targets are needed for restless legs syndrome. Preclinical data suggest that a hypoadenosinergic state plays an important pathogenetic role.

**Objective:**

The objective of this study was to determine whether inhibitors of equilibrative nucleoside transporters, for example, dipyridamole, could provide effective symptomatic treatment.

**Methods:**

A 2‐week double‐blind, placebo‐controlled crossover study assessed the efficacy of dipyridamole (possible up‐titration to 300 mg) in untreated patients with idiopathic restless legs syndrome. Multiple suggested immobilization tests and polysomnography were performed after each treatment phase. Severity was assessed weekly using the International Restless Legs Rating Scale, Clinical Global Impression, and the Medical Outcomes Study Sleep scale. The primary end point was therapeutic response.

**Results:**

Twenty‐eight of 29 patients recruited were included. International Restless Legs Rating Scale scores improved from a mean ± standard deviation of 24.1 ± 3.1 at baseline to 11.1 ± 2.3 at the end of week 2, versus 23.7 ± 3.4 to 18.7 ± 3.2 under placebo (*P* < 0.001). Clinical Global Impression, Medical Outcomes Study Sleep, and Multiple Suggested Immobilization Test scores all improved (*P* < 0.001). The mean effective dose of dipyridamole was 217.8 ± 33.1 mg/d. Sleep variables improved. The mean periodic leg movement index at the end of treatment with dipyridamole was 8.2 ± 3.5 versus. 28.1 ± 6.7 under placebo. Side effects (dipyridamole vs placebo) included abdominal distension (18% vs. 7%), dizziness (10.7% vs 7.1%), diarrhea, and asthenia (each 7.1% vs 3.6%).

**Conclusions:**

Dipyridamole has significant therapeutic effects on both sensory and motor symptoms of restless legs syndrome and on sleep. Our findings confirm the efficacy of dipyridamole in restless legs syndrome predicted from preclinical studies and support a key role of adenosine in restless legs syndrome. © 2021 The Authors. *Movement Disorders* published by Wiley Periodicals LLC on behalf of International Parkinson and Movement Disorder Society

Although dopamine agonists are effective agents for the short‐term treatment of restless legs syndrome (RLS)/Willis‐Ekbom disease, their main complication during long‐term treatment is the augmentation of RLS symptoms, which manifests as an overall increase in symptom severity.^1^ If not stopped in time, augmentation can progress, becoming a serious complication that leads to treatment discontinuation. Once established, its management is complicated by the symptoms not responding well to standard alternative agents.^2^ Studies show that after a treatment period of approximately 10 years, the prevalence of augmentation nears 50%.^3^, ^4^, ^5^ Furthermore, given that RLS is frequently a chronic disease, it is likely that with protracted treatment the risk of augmentation will increase even further.^6^ In light of this, there is a clinical need for treatment alternatives to dopaminergic drugs.

A recent open study has shown that the nonselective blocker of equilibrative nucleoside transporter (ENT) dipyridamole significantly improves RLS dysesthesias, periodic limb movements (PLMs), and sleep during 8 weeks of treatment.^7^ The efficacy of dipyridamole was predicted from pre‐clinical evidence of a brain iron deficiency (BID)–induced hypoadenosinergic state.^8^, ^9^ Thus, BID is considered a main initial pathophysiological mechanism in the development of RLS.^10^, ^11^ Also, preclinical studies have shown that BID in rats leads to striatal downregulation of adenosine A_1_ receptors (A1Rs),^10^ which could explain the increased sensitivity of corticostriatal glutamatergic terminals observed in the same RLS animal model.^12^ It is significant that the BID‐induced increase in sensitivity of corticostriatal terminals could be reproduced by local application of an A1R antagonist and could be counteracted by dipyridamole.^9^ Dipyridamole is a nonselective inhibitor of equilibrative nucleoside transporters ENT1 and ENT2, thereby increasing extracellular adenosine,^13^ and its ability to promote activation of striatal adenosine receptors upon its systemic administration was recently demonstrated in reserpinized mice.^14^

We report here on the first placebo‐controlled clinical trial evaluating, under double‐blind conditions, the efficacy of dipyridamole for sensory and motor symptoms in idiopathic RLS, as measured by subjective rating scales (primary objective). In addition, we aimed to evaluate its potential therapeutic effects as measured by the Multiple Suggested Immobilization Test (m‐SIT) and polysomnography, along with a first estimate of the therapeutic dose range in RLS, its tolerability, and possible toxic effects.

## 1 Methods

The study was performed at the Sleep Research Institute, Madrid, Spain, and approved by the local institutional review board. Written informed consent was obtained from all participants.

The study was performed as a randomized, double‐blind, placebo‐controlled crossover clinical trial during which 28 previously untreated patients diagnosed with idiopathic RLS were treated for 2 consecutive 2‐week periods with dipyridamole and placebo.

Patients met the 2014 International Restless Legs Syndrome Study Group diagnostic criteria for idiopathic RLS,^15^ had an International Restless Legs Rating Scale (IRLS) score at baseline greater than 20, and suffered symptoms at least 3 times per week. Recruited patients were not suffering from augmentation^16^ and lacked significant RLS symptoms before 7:00 pm. None of the patients included had been previously treated with dipyridamole. Following a 2‐week washout period for any other psychoactive substance, the drug (placebo or dipyridamole) was administered for the first 3 days at a dose of 100 mg/d, then up‐titrated to 200 mg/d, and if considered clinically necessary, was increased to 300 mg/d during the second week. Following a 1‐week washout period, patients started another 2‐week treatment period with the alternative treatment regimen. A computerized randomization list was used to allocate patients to each treatment order on a 1:1 basis. The staff in charge of the list were not in contact with the patients and did not influence the order of treatment, which was transmitted by a sealed envelope. Masking of the medication was achieved using tablets identical in appearance to the active substance and placebo. Participants, research staff involved in treatment or assessment of outcomes, and those involved in the data analysis were at all times blinded to group assignment.

Study medication was administered at 9:00 pm. Patients were instructed to abstain from any coffee after 2:00 pm during the 2 weeks prior to study initiation.

Assessment of severity was performed every 2 weeks using the IRLS (primary end point) and the Clinical Global Impressions‐S (CGI) scale and the Medical Outcomes Sleep Scale (MOS), both secondary end points.

Furthermore, at the end of each treatment period, an m‐SIT followed by polysomnography was performed (both also secondary endpoints). The m‐SIT is a validated test and is frequently used in proof‐of‐concept studies because of its high sensitivity to signal detection, thereby allowing a reduction in the required number of participants.^17^ It evaluates the severity of motor and subjective RLS symptoms using a numerical symptom severity scale while the patient is awake and immobile for 60 minutes at 8:00 pm, 10:00 pm, and midnight. Leg movements were measured by surface electromyography monitoring of both anterior tibialis muscles. Polysomnography was performed between 1:00 am and 7:00 am and scored according to standard American Academy of Sleep Medicine criteria.

Side effects were assessed weekly using a checklist including the most common side effects of dipyridamole.

### 1.1 Statistical Analysis

Calculation of the sample size was based on the results of a similar 2‐week crossover study.^18^ According to this calculation, with 25 patients per treatment condition there is at least 80% power to detect a difference of at least 4 points between the treatment changes in dipyridamole and placebo. The test assumed a type I error of 0.025 with 1‐sided testing.

All efficacy analyses were carried out using the intention‐to‐treat population (ITT), which was defined as all patients who received randomized treatment (covers International Conference on Harmonisation E6, section 6.7.2).

The Kolmogorov–Smirnoff test was used to evaluate the normality of distributions. Paired‐sample tests were used to analyze dependent variables (primary and secondary end points), with the paired *t* test used if distribution was normal and the Wilcoxon test used if not. Because of the experimental character of the study and the sample size, the significance level used was 0.05, with mention of any values below 0.1.

## 2 Results

**FIG. 1:**
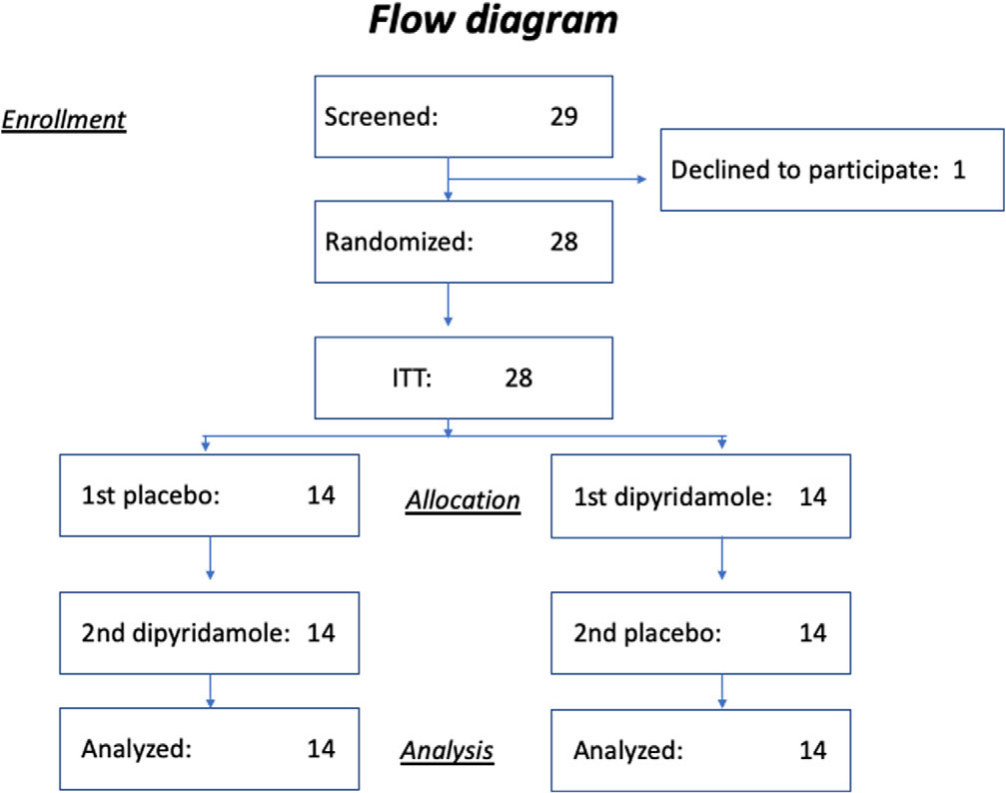
Consort flow diagram. [Color figure can be viewed at wileyonlinelibrary.com]

Recruitment was performed between April 2018 and March 2020. Twenty‐nine subjects were screened, 28 of whom were randomized and finished the study (see Fig. 1). Table 1 shows the patients’ main clinical and demographic characteristics.

**TABLE 1:** Main demographic and clinical variables

Age (y)	60.25 ± 7.09
Sex (% women)	65
Race (% white)	100
BMI	29.05 ± 3.53
Concomitant disease, n (%)	
Cardiovascular	8 (40%)
Osteoarticular	2 (10%)
Hepatobiliary	2 (10%)
Psychiatric	2 (10%)
Dermatological	1 (5%)
Gastrointestinal	1 (5%)
Hyperacusia	1 (5%)
Concomitant medication, n (%)	
Insulin	0 (0%)
Nonsteroid anti‐inflammatories	17 (85%)
Analgesics	16 (80%)
Cardiovascular	8 (40%)
Gastrointestinal	8 (40%)
Antiprostatics	3 (15%)
Endocrine	4 (20%)

### 2.1 Efficacy Variables

**FIG. 2:**
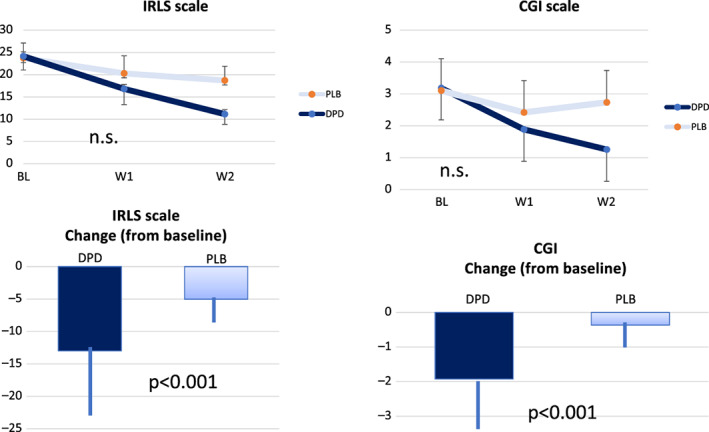
RLS severity scales (IRLS, CGI) across treatment conditions. [Color figure can be viewed at wileyonlinelibrary.com]

IRLS (24.1 ± 3.1 vs 23.7 ± 3.4, not signicant) and CGI (3.2 ± 0.9 vs 3.1 ± 0.9, not significant) scores at baseline were similar across treatment conditions. However, change following treatment with dipyridamole was significantly greater than with placebo (mean ± SD for IRLS: −13 ± 0.8 vs −5.0 ± 0.2, *P* < 0.001; for CGI: −1.9 ± 0.3 vs −0.4 ± 0.2, *P* < 0.001; Fig. 2).

**FIG. 3:**
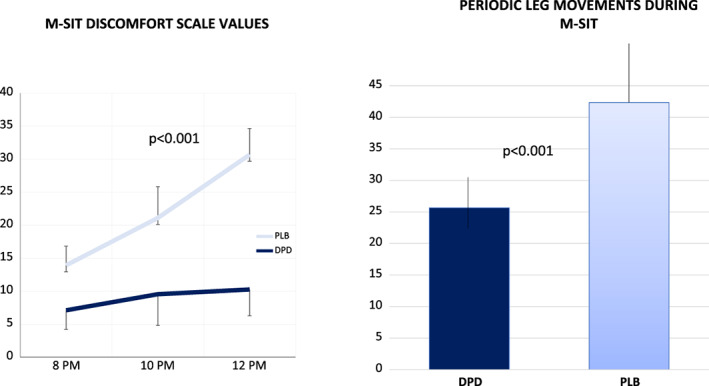
Results of the Multiple Suggested Immobilization Test across treatment conditions. [Color figure can be viewed at wileyonlinelibrary.com]

Similarly, there were greater improvements for dipyridamole than for placebo on the m‐SIT, both on the subjective scale (mean ± SD percent of maximal score m‐SIT‐ds: 15.6 ± 8.6 vs 28.3 ± 7.2, *P* < 0.001) and on motor dysfunction (25.6 ± 8.6 vs 42.3 ± 7.2, *P* < 0.001; Fig. 3).

### 2.2 Sleep

MOS‐sleep scale scores improved under dipyridamole compared with placebo in the subscores sleep adequacy (*P* < 0.001) and sleep quantity (*P* < 0.001), but there were no differences in daytime somnolence between groups according to the MOS or the Epworth Sleepiness Scale.

Results of the sleep studies showed improvements favoring dipyridamole in total sleep time, sleep latency, wake after sleep onset time, sleep efficiency, arousal index, and stage N3 (Table 2).

**TABLE 2:** Mean ± SD values for the main polysomnographic variables

	Dipyridamole		Placebo		*P*	Effect est.	95% CI
	Mean	SD	Mean	SD			
Total recording time (min)	463.1	32.7	450.7	44.6	0.273	12.37	−10.32 to 35.06
Total sleep time (min)	382.2	45.1	351.1	56.3	0.014	31.11	6.92‐55.29
Sleep latency (min)	20.3	8.1	27.5	11.1	**0.007** ^a^	−722	−12.07 to −2.38
Sleep efficiency (%)	82.5	5.9	77.1	7.9	**0.003** ^a^	5.41	2.26‐8.56
Wake after sleep onset (min)	59.8	22.9	74.3	30.3	0.018^b^	−14.48	−26.32 to −2.65
Arousal index (#/h)	23.5	15.1	30.7	14.2	0.020^b^	−7.18	−14.21 to −0.15
N1 (min)	61.1	29.6	61.4	28.1	0.633	−0.34	−15.77 to 15.10
N2 (min)	207.1	31.6	202.7	51.7	0.619	4.30	−13.27 to 21.87
N3 (min)	55.3	16.1	33.2	20.9	**< 0.001** ^a^	22.17	12.91‐31.42
REM (min)	58.0	30.0	54.0	28.5	0.559	4.01	−9.90 to 17.92
N1 %	16.1	7.5	18.3	9.1	0.314	−2.11	−6.32‐2.11
N2%	54.3	6.9	57.3	9.5	0.077	−2.89	−6.12 to 0.33
N3%	14.5	3.7	9.4	5.6	**< 0.001** ^a^	5.11	2.83‐7.38
REM, %	14.8	6.4	15.1	7.0	0.840	−0.32	−3.56 to 2.92
REM latency (min)	105.0	38.7	107.4	40.1	0.803	−2.43	−22.23 to 17.37
HR (bpm), mean	62.3	4.2	63.7	4.3	0.194	−1.39	−3.53 to 0.75
SaO_2_ baseline (%)	94.1	1.4	94.2	1.4	0.799	−0.08	−0.72 to 0.56
SaO, mean (%)	92.3	1.4	92.6	1.5	0.316	−0.34	−1.02‐0.34

^a^ *P* < 0.01.

^b^ *P* < 0.05.

In addition, compared with placebo, dipyridamole further reduced both the frequency of PLMs of sleep (PLM index: 7.5 ± 4.0 vs 19.1 ± 8.2) and PLMs associated with arousal (PLM–arousal index: 2.5 ± 1.5 vs 11.2 ± 6.5; *P* < 0.001); see Table 2.

### 2.3 Dose

Mean ± SD dose at the end of the dipyridamole treatment period was 218 ± 33.1 mg/d with 18% of the patients needing 300 mg/d. In contrast, the mean dose in the placebo group was 250 ± 50.0 mg/d (*P* < 0.05).

### 2.4 Side Effects

The main side effects under dipyridamole (compared with placebo) were abdominal distension (18% vs 7%), dizziness (10.7% vs 7.1%), diarrhea, and asthenia (both 7.1% vs 3.6%). All other side effects (daytime somnolence, ataxia, dry mouth, edema, and pruritus) were below the 5% threshold. These side effects were mild, and none led to study discontinuation.

## 3 Discussion

Our results suggest that compared with placebo the nonselective ENT1/ENT2 inhibitor dipyridamole at a mean dose of 218 mg/d improves RLS sensory and motor symptoms and sleep over a treatment period of 2 weeks. This finding is supported not just by RLS evaluation based on rating scales (given the subjective nature of most RLS symptoms), but also by objective tests such as sleep studies and the m‐SIT. The drug was in general well tolerated, with no patients discontinuing during treatment. In addition, as could be expected from a drug that increases extracellular adenosine in the brain,^19^ dipyridamole increases sleep time and slow‐wave sleep and reduces wake‐time both before and during sleep.

These findings confirm the results of a previous open‐trial study in which dipyridamole improved RLS symptoms over an 8‐week treatment period.^7^ Our study has several limitations, which are the small sample size and the short treatment period of 2 weeks. Nevertheless, its first objective was to provide proof‐of‐concept results showing a significant therapeutic response to a drug with a new mechanism of action. Furthermore, because all the patients included were naive to dopaminergic treatments, a factor that has been shown to exert an influence on the therapeutic outcome of nondopaminergic agents,^2^ its usefulness in patients resistant to dopamine agonists or patients undergoing augmentation needs to be determined.

These findings could not only pave the way to a new therapeutic approach for RLS but might also support preclinical evidence obtained in animal models of RLS that indicate the existence of a hypoadenosinergic state in RLS (see introductory section). BID constitutes a well‐accepted initial pathogenetic mechanism in RLS.^11^ Based on results obtained in the BID rodent model of RLS, it has been recently hypothesized that a hypoadenosinergic state constitutes the next link in the pathogenetic chain of events, mostly secondary to downregulation of A1Rs in the brain,^8^, ^9^, ^10^, ^14^ which predicted that promoting an increase in the extracellular concentration of adenosine in the central nervous system would be beneficial for the symptomatic treatment of RLS.

It is well established that in the brain, adenosine uptake from the extracellular space occurs primarily by facilitated diffusion through equilibrative transporters and that their inhibition leads to an increase in the extracellular concentration of adenosine.^20^, ^21^, ^22^ From the 4 subtypes of equilibrative transporters so far identified, ENT1 and ENT2 are the most expressed in the brain, mostly in astrocytes, but also in neurons.^22^ Dipyridamole is a nonselective inhibitor of ENT1 and ENT2, and it still needs to be determined which subtype is the main target responsible for its therapeutic effects. New molecules need to be developed and investigated preclinically to better dissect the main therapeutic target of dipyridamole. Furthermore, ENT1/ENT2 inhibitors are one strategy to increase extracellular adenosine levels. The role of other strategies, such as inhibitors of adenosine metabolization or ketogenic diets,^23^ should also be clinically evaluated.

## Author Roles

Diego Garcia‐Borreguero: study concept and design, study supervision, acquisition of data, first draft of article.

Celia Garcia‐Malo: study concept and design, first draft of article.

Juan José Granizo: statistical analysis and interpretation of data, critical revision of manuscript for intellectual content.

Sergi Ferré: study concept and design, first draft of article.

## Financial Disclosures

Diego Garcia‐Borreguero: employed by Sleep Research Institute, Madrid; grants from MSD. Celia Garcia‐Malo: employed by Sleep Research Institute, Madrid. Juan José Granizo: employed by Instituto de Investigaciones Sanitarias Puerta de Hierro, Madrid. Sergi Ferré: employed by NIDA; intramural funds from the National Institute of Drug Abuse.
